# Dentistry

**Published:** 1874-08

**Authors:** P. H. Austen


AMERICAN JOURNAL

OF

DENTAL SCIENCE.

Vol. VIII. THIRD SERIES--AUGUST, 1874. No. 4.

### ARTICLE I.

### *Dentistry.*

BY P. H. AUSTEN, M. A., M. D., D. D. S.

Address delivered before the American Academy of Dental Science, at their Sixth Annual Meeting, held in Boston, September 29th, 1873.

*Mr. President and Fellows of the American Academy of Dental Science:*—MEDICINE is as old as human disease, which it seeks to cure; and MECHANISM dates as far back as the wants of mankind, to which it ministers.

Youngest born of these two is DENTISTRY, bearing more distinctly than any known art the impress of its double lineage. For what branch of Medical Art so dependent upon mechanical genius; and what branch of Mechanism so directly addressed to the relief of those ills which human flesh is heir to?

The childhood of Dentistry has been unfortunate, in that, while disowned by one parent, it has been taught to look down upon the other. For Medicine, in all ages, has been prone to despise Hand-craft, as beneath the attention of those who claim that, by pure might of intellect, they can conquer the many-headed hydra—Disease. But medical practice is no longer the unit it once was; and the "Family Physician" is soon destined to become an institution of the past.

As the centuries roll on, the boundaries of Science and Art are enlarged, but the measure of individual capacity remains the same. Hence, Medical Practice divides and sub-divides, in order to the fullest development of its several departments.

The physician has no longer time for Pharmacy or Surgery; nor dare he hereafter affect to despise Arts quite as essential to life as his own advice and prescription. Even this advice, to be most effective, grows out of the special study of separate organs.

Thus the Body Medical, like the human body which it studies, is composed of many members. "The eye can no longer say to the hand, I have no need of thee;" for so close is this union, that when one member suffers, all must suffer; and when one is honored, all should rejoice. Why, in this great family of the Medical Arts, is young Dentistry so neglected and excluded from the family circle? Partly because he don't like his books, and this family is proud of its intellect and high education; partly because he is too fond of his grandfather's workshop.

The first is a grave fault, to be hereafter noticed; but is the second a just ground of reproach? Shall we stigmatize Dentistry, born in this 19th century—the century of invention—for having so large an element of mechanism? Gentlemen of this Academy, guardians of Dentistry, (as yet a minor,) do you also share in this one-sided pride of lineage? If Mechanism, *per se*, is discreditable, so is Dentistry; for it is its very life blood. As well might a man be ashamed of his own mother.

I have elsewhere divided Dental Art into Medical, Surgical, and Prosthetic. The two first connect it with the healing art, and demand a medical education; but the characteristic element of Dentistry is its Prosthetics,—just as Therapeutics is the specific function of the Physician. To remove diseased structure, and replace it with gold,—to remove diseased organs, and replace them with porcelain,—is the work which demands nine-tenths of the dentist's time; success in which gives him his reputation.

You can call the one Operative Dentistry, and the other Mechanical Dentistry, if you choose; but each consists in a series of operations, and both are purely mechanical manipulations of material, by means of instruments; both, also, are acts of replacement. I think it, therefore, more exact and descriptive to subdivide the peculiar work of Dentistry into—Structural and Organic Prothesis.

Both are so difficult, that highest excellence in either department is rare, and scarcely ever do we meet with a "double first class." Hence, the practice of Dentistry is itself subdivided, following the example of its parent art. But subdivision does not imply less honor in the pursuit, so long as we recognize, in preparation for it, the necessity of a knowledge of the whole art of which it forms a part.

This brings us to the only valid objection against the recognition of Dentistry as a specialty of Medicine. If it be true that dentists, as a class, have a more defective education than other specialists; if it be true that a large number of recognized members of the Dental profession have no medical education whatever, there is good reason for this hesitancy.

Is Dentistry, then, a LIBERAL profession? Yes, certainly, if the majority of its members are men of liberal education. Medicine numbers among its practitioners very many half-educated and not a few wholly ignorant men. But such are not the men who to-day control that profession; or who, in the past, have given it dignity and reputation.

I have already intimated that Dentists are too prone to spend in mechanical details time which should be given to study, and to adopt the popular error that a "mechanical turn" is the one grand element of success. It is indeed a

*sine qua non*, without which the selection of the Dental branch of medicine would be a sad waste of effort. But skill without education, art without science, cannot be called a Profession—I mean in the modern sense in which that term is applied to Law, Medicine, and the Ministry.

How shall we separate from the mass of those who call themselves Dentists such as may justly claim to be members of the profession of Dentistry, and, by virtue of this claim, members also of the Medical profession? This is the most imperative, as it is the most difficult, duty which to-day lies before this Academy. Effort in this direction must be co-operative: it must also be harmonious.

Personally you are each responsible for your *individual* reputation; personally, however, you can do no more than add a unit to the *collective* reputation of the profession. But, by associate action, you can decide who shall unite with you in establishing a general professional character.

Dental societies, associations, and academies have heretofore suffered other and less important objects to engross the hours of conference. Undoubtedly much good has been done by such meetings. But to what purpose do you improve the field of your labors unless you first enclose it, and have a well guarded entrance? What harvest can be gathered on an open common?

Gentlemen, I call upon you, first of all, to establish your metes and bounds, and enclose your domain; for then, and only then, can you hope to reap the fruit of your toil. Then, with some hope of general adoption, can you frame a code of professional ethics, and encourage gentleman to enter the profession by guaranteeing them the courtesy due to gentlemen. Then can you establish a higher standard of work than cheapness, and bring about a more generous rivalry than underbidding and defamation. For you well know that there is a large class whose actions, unnecessary to be here specified, greatly damage the character of the profession which it is your pride to honor. You must exclude or reform them—and that by half-way measures—or you must fall to their level.

You must also establish a Dental Literature. I do not mean text-books, although these might be increased in number, and, in some departments of the art, greatly improved. I do not refer to the so-called Dental journals, which, for the most part, are chiefly advertising media of depots or colleges. Now and then we find in them an excellent article; but alas! what an iteration, *ad nauseam*, of experiences, which a little more reading, study, and general education might have spared both writer and readers!

I speak of thoughtful and well written monographs and treatises, which shall not only interest the dental, but command the notice and approval of the medical profession; articles, showing that there are able and experienced men in your profession willing to spend some hours for its advancement, not measured by the golden rule of the operating chair.

Making ample allowance for difference in the number of physicians and dentists, we are forced to the conclusion that Dentistry is the least literary of all the departments of Medicine. Let us charitably attribute this to the modesty of a young profession, and hope for better days.

A much neglected yet most important element of Dental Art is its Æsthetics. It is a fact, much to the discredit of the profession, that many forms of great beauty in ceramic art lie in dental depots unsought for, because of the incapacity of dentists to appreciate and use them. Thus artistic genius is repressed in its efforts to benefit Dentistry, and the Art itself suffers in reputation, because it seems to be incapable of what it can really accomplish. Take this in connection with one other fact—that second and third rate apparatus, implements, and materials find more ready sale than first class and higher priced ones—and we are brought to the melancholy conclusion that not only is there too little science among dentists, but that the much boasted "Art and skill" which is to take precedence of all other qualifications, is really not of the highest order.

I gravely doubt if the average mechanical skill exercised in dental offices and laboratories would be tolerated for a day in any machine-shop in the land. When I said that in mechanism *per se* there was nothing degrading, I referred to no such work as this. For there is here an incompetence or a neglect which has nothing to excuse or redeem it, and which is, in the highest degree, disgraceful. It argues nothing against the Dental profession to condemn such workers, for they do not belong to it.

I have reserved for final consideration the duty of your Academy in the matter of Dental Education; for in this work the Academies and Societies of the profession must take the lead,—the Colleges play only a subordinate part, however important. Misapprehension on this point has led some of the best men of the profession to censure our colleges with undue severity.

They have done great good, and their teachers have generously given a vast amount of time, thought, and labor to the cause of education. I say *given;* for the compensation, as compared with that awarded to dental services, has ever been paltry, and has often been in the form of actual loss. Had those who blame been half so faithful to their office students, as college professors to theirs, the schools would have had better material to deal with. Had societies enforced compliance with the standard of the colleges, low as it is, we should to-day have had a far higher standard of professional education. As it is, the better half of the young men of the profession owe more to the colleges, than to any other single influence.

Although therefore not failures, in that they have done much good, yet must we write on the walls of our colleges the sad word "TEKEL." They have been "weighed in the balances, and found wanting," not only because unsustained, but because organized after the model of American *Medical* schools. Medicine gains no honor through the average medical graduate; and more credit is given to the average dental graduate, only because so many dentists lack even that amount of preparation.

The American physician supplements the defect of his education by walking the hospitals of England and Europe.

But American dentistry so far excels the transatlantic in her Prosthetics, that Europe comes here to learn. Where, then, can we go to make up our shortcomings in the other branches of Dental Education? I answer: by remodelling the entire system of dental instruction.

All Dental Colleges south of Boston are organized upon the plan of the Baltimore College of Dental Surgery, to whose principal founder, President CHAPIN A. HARRIS, the profession is so greatly indebted. You will, I trust, acquit me of disparagement or disrespect to my college, the *Alma Mater* of some members of this Academy, if I tell you what I think are the grave defects of this organization, as time and experience have revealed them.

First, then: it receives students without preliminary examination. No literary college does this; and no professional school can do it, without gross injustice to itself. It is hard enough to be compelled to crowd four years' teaching into eight months. But when the recipients of this teaching have no trained habits of study; know nothing of the first elements of science, and have not even such slight knowledge of Latin and Greek as enables them to understand the necessary technicalities of Medicine,—then is the work of instruction worse than Egyptian Bondage; it is, truly, "making bricks without straw."

Secondly: it examines for graduation after TWO terms of study. So much has been said upon this point, that I shall dismiss it with one remark. The profession that tolerates, for its raw recruits, less than four years of diligent study (mark me, I do not mean simply four courses of lectures,) must be content to allow its colleges to send ont many graduates imperfectly prepared to enter its ranks.

Thirdly: the Faculty are the Examiners for graduation. One of three evils is unavoidable. The professor must hold himself sternly aloof from his pupils, thus loosing one of the most effective aids to his teaching—the friendly word of advice and encouragement. Or he must do grievous violence to his feelings by rejecting those, whose struggling progress he has watched and aided with such interest. Or he must risk the character of the profession and of his school by giving honor to those unworthy of it.

No teacher should be placed in this dilemma. The English examiner gets handsomely out of it, by retiring from the room, if the student chances to be even socially and ever so slightly known to him. I commend the English custom for American adoption, well satisfied that, until examiners and teachers are totally distinct bodies, no diploma can be quite clear from suspicion of partiality.

It is said that Faculties are afraid to be rigid in examination, for fear students will prefer a more lenient school. The sooner such a school ceases to have graduates the better. But gentlemen, in justice to students, permit me to give, as the result of my twenty years' experience, that all students, who deserve the name, respect and love most those teachers who put them through the severest drill.

To the Boston Faculty of Dentistry I tender this word of advice: Be as radical in your profession as you were in your politics. Refusing to compromise with slavery, you, with a high hand, abolished it. Do not, then, be yourselves slaves to the past, through timid fear of the consequences of radical innovation. As Alumni of the Baltimore School, give your Alma Mater all honor for what she has done, but do not copy her mistakes.

Thirty-three years is an average human life, but a very short period in the existence of a profession. Dental education may, without shame, confess the errors of its infancy, especially if this confession throws light upon the pathway of the future. The new experiment can come from no city with better grace, than from the modern Athens.

May I, gentlemen of the College and the Academy, wishing heartily success to your efforts, offer for your consideration a few parting words.

Under the shadow of old Harvard, do not make your connection with her Faculty a pretence and a sham by accepting students, who have not pursued an honorable course of literary study.

Working in harmony with the Medical School, do not dishonor your specialty by accepting from your graduates any lower grade of medical knowledge, than is required for the oculist, aurist, or general surgeon.

Build up for Dentistry what other departments of Medicine possess in their magnificent hospitals, asylums, and infirmaries. For medical education is rapidly resolving itself into clinical instruction in specialties. The time, I think, is not very distant when a rigorous examination in the science of general medicine will be demanded as essential to admission to the wards of all hospitals for specific diseases, and when years spent in the best of such institutions will be the only recognized qualification for the practice of any medical specialty.

If the College will thus acquit itself, and the Academy will labor in connection with other societies in the States for the establishment of a "Supreme Court," whose decisions as to professional character shall be final—Dentistry will enter upon its manhood under auspices which will attract her right proportion of the genius, talent, and energy of the country, and will reflect back upon her members the honor and dignity which she receives from them.

